# Congenital eyelid ptosis, decreased glomerular filtration, and orthostatic hypotension: Answers

**DOI:** 10.1007/s00467-016-3515-1

**Published:** 2016-11-17

**Authors:** Tessa Wassenberg, Michèl Willemsen, Henry Dijkman, Jaap Deinum, Leo Monnens

**Affiliations:** 1 Department of Neurology and Donders Institute for Brain, Cognition and Behaviour, Radboud University Medical Center, PO Box 9101, 6500 HB Nijmegen (935), The Netherlands; 2 Department of Pathology, Radboud University Medical Center, Nijmegen, The Netherlands; 3 Department of Internal Medicine, Radboud University Medical Center, Nijmegen, The Netherlands; 4 Department of Physiology, Radboud University Medical Center, Nijmegen, The Netherlands

**Keywords:** Orthostatic hypotension, Dopamine beta hydroxylase deficiency, Proximal tubule

## What diagnosis can be suspected and which tests can be used to confirm this?

**Fig. 1:**
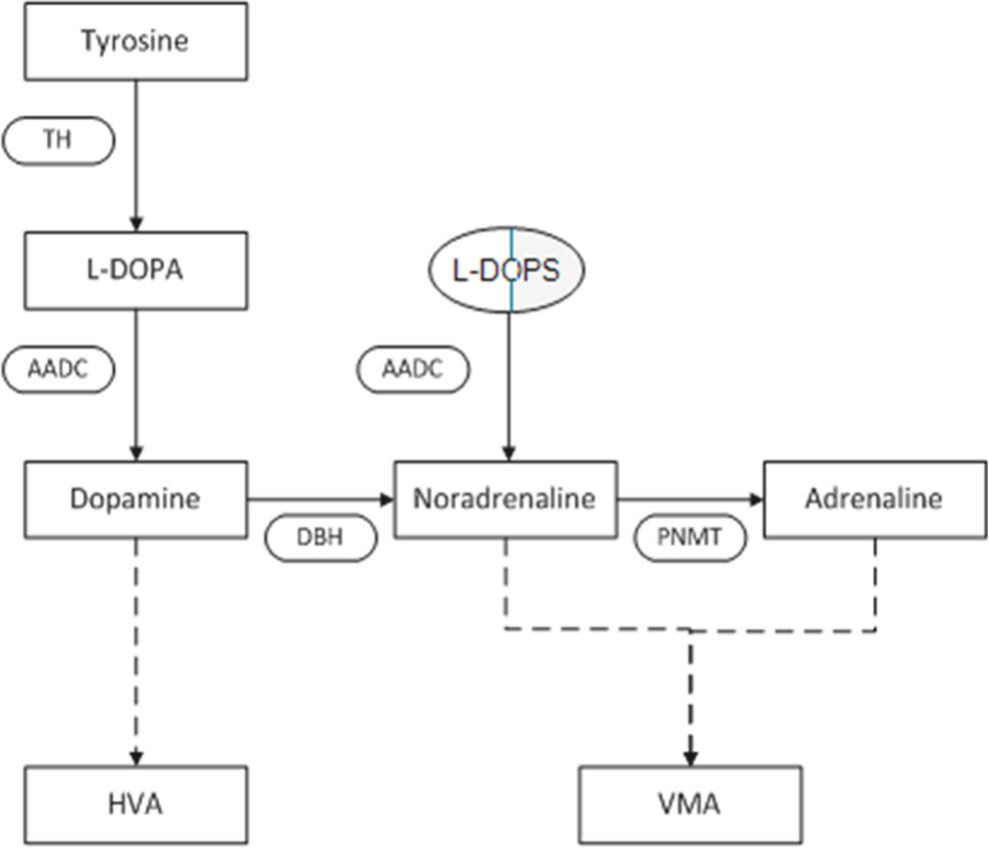
Simplified scheme of catecholamine biosynthesis. The diagram shows how catecholamines (dopamine, noradrenaline, and adrenaline) are formed from tyrosine and L-DOPA, and how they are metabolized to homovanillic acid (*HVA*) and vanillylmandelic acid (*VMA*). Enzymes, represented in ovals, are abbreviated: *TH* tyrosine hydroxylase, *AADC * aromatic l-amino acid decarboxylase, *DBH* dopamine beta hydroxylase, *PNMT* phenylethanolamine N-methyltransferase. The metabolic block in DBH deficiency can be bypassed by supplying the patient with L-DOPS, represented in a capsule. *Dashed arrows* do not show intermediate steps/enzymes

The combination of congenital eyelid ptosis and severe orthostatic hypotension with preserved heart rate acceleration suggests sympathetic failure with intact parasympathetic function. Sweating, sympathetically mediated by acetylcholine, was intact, which implies that there was isolated absence of noradrenergic sympathetic function. Indeed, noradrenaline and adrenaline in plasma at rest and after standing were below detection limits (<0.01 nmol/L, normal values 0.4–3 nmol/L and 0.012–0.2 nmol/L respectively), whereas the dopamine level was greatly increased (1.1 nmol/L, normal values 0.02–0.19 nmol/L). These very low plasma levels of noradrenaline and adrenaline with an increased level of dopamine in this young adult patient are typical of dopamine beta hydroxylase (DBH) deficiency. DBH deficiency in this patient was genetically confirmed, with homozygous known pathogenic variants (IVS1 + 2 T > C) of the *DBH* gene (chromosome locus 9q34.2) [1]. As shown in Fig. 1, DBH is the enzyme that converts dopamine to noradrenaline. Noradrenaline can then be further converted to adrenaline. DBH deficiency is an extremely rare metabolic disorder of catecholamine synthesis, described in less than 20 patients worldwide. It leads to a lack of sympathetic noradrenergic function with normal parasympathetic and cholinergic-mediated sympathetic function, as is extensively reviewed by Robertson and Garland [2]. In DBH deficiency, the autonomic nervous system is structurally intact, but functionally impaired by the lack of noradrenaline, which leads to profound orthostatic hypotension and mild eyelid ptosis, but has no effect on sweating. In most patients, baroreflex afferents to the heart are at least partially intact, as demonstrated by the increase in heart rate on standing [3].

In DBH deficiency, it is described that orthostatic hypotension mostly becomes manifest during adolescence. The reason for this relatively late presentation in the context of severe catecholamine deficiency from birth onward is unknown. It may be partly due to tardy recognition, as was probably the case in our patient. In general, orthostatic intolerance is often considered and evaluated in puberty and adulthood, but not in childhood. We are not aware of studies on normal values for blood pressure after orthostatic provocation in young children. Moreover, orthostatic complaints may become more apparent during puberty, because children may be protected from it owing to reduced gravitational force on their limited body height, or because of increased angiotensin II levels in childhood. Angiotensin II is part of the neurohumoral response on standing [4].

## What could be an explanation for the mitochondrial abnormalities in the proximal tubule, and the decreased glomerular filtration rate in this patient?

**Fig. 2:**
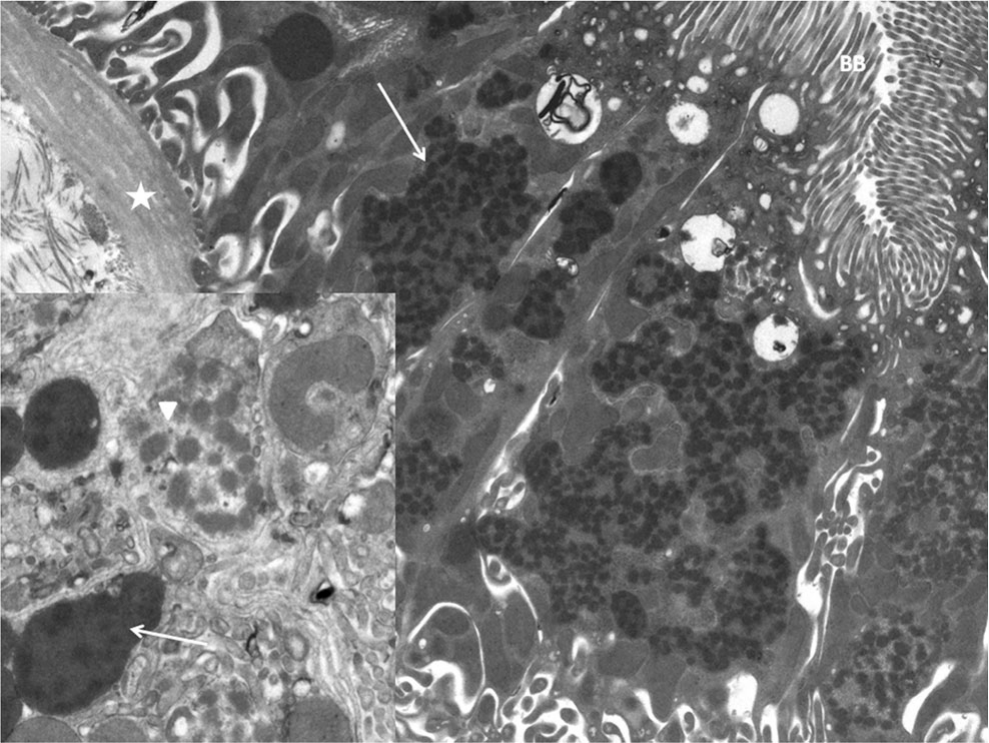
Electron microscopy of kidney biopsy showing notable abnormal mitochondria. *Star* basal membrane, *BB* brush border proximal tubule. *Inset*: swollen mitochondria exhibit electron-lucent areas (*arrowhead*), fused mitochondria present as amorphous matrix densities (*arrow*)

Electron microscopy of the kidney biopsy (Fig. 2) showed notable mitochondrial abnormalities, with extensive mitochondrial fusion and mitochondria with irregular shape and/or loss of cristae. This indicates mitochondrial dysfunction, especially in the activity of fission and fusion, which is critical for preserving normal cellular physiology [5], and can be an indicator of changed environmental conditions. No histological abnormalities were seen at the distal tubules, and mitochondrial function in muscle tissue was normal, suggesting a local damaging factor in the proximal tubule in our patient. A possible damaging factor here is the high L-DOPA concentration in proximal tubules, because of increased filtered load and enhanced transport of L-DOPA in this compartment [6]. In DBH deficiency, plasma L-DOPA concentration is doubled [1, 3]. L-DOPA is metabolized to dopamine inside the proximal tubular cells and dopamine is transported out of the cell to activate dopamine receptors. In urine, the concentration of dopamine is about 1,000 times the serum concentration and a small increase in L-DOPA concentration can lead to greatly increased dopamine levels [7]. Goldstein et al. reviewed the inherent cytotoxicity of catecholamines including dopamine in cells in which they are produced [8, 9]. Monoamine oxidase (MAO), one of the enzymes that metabolize dopamine to its inactive metabolite homovanillic acid, yields hydrogen peroxide, resulting in the production of reactive oxygen species (ROS). Increased production of mitochondria-derived ROS was shown in opossum proximal tubular cells treated with dopamine in a concentration-dependent manner [10]. Mitochondrial DNA is particularly susceptible to modification by ROS [11]. Furthermore, mitochondrial toxicity can be due to increased levels of quinones, which are intermediates in L-DOPA metabolism [12].

Besides mitochondrial dysfunction, the function of the tubuloglomerular system itself may be an explanation for the decreased eGFR in our patient. Dopamine leads to decreased reabsorption of sodium in the proximal tubule and loop of Henle, and this could initiate a vasoconstrictor response in the afferent arteriole via a Na^+^-K^+^-2Cl^−^ cotransporter, and a vasodilator response, probably via the Na^+^ transporter in the connecting tubule. Both transporters, however, are inhibited by dopamine. Dopamine can also directly stimulate renin expression and release [13]. It is difficult to predict the effect of increased dopamine on glomerulotubular balance. Other factors, caused by defective reabsorption by damaged proximal tubular cells such as succinic acid, can stimulate the system [14]. However, dopaminergic stimulation of the tubuloglomerular feedback system leading to reduced filtration pressure remains an attractive possibility.

Decreased kidney function was found in 4 out of 9 patients with DBH deficiency [2]. Garland et al. proposed that a decrease in renal perfusion may lead to renal damage in patients with autonomic failure [15]. However, if hypoperfusion leading to hypoxia would be the main explanation of kidney failure in this patient group, tubulointerstitial fibrosis would be expected [16]. Fibrosis was only discreetly seen in our patient (Fig. 2).

## Is treatment possible?

**Fig. 3:**
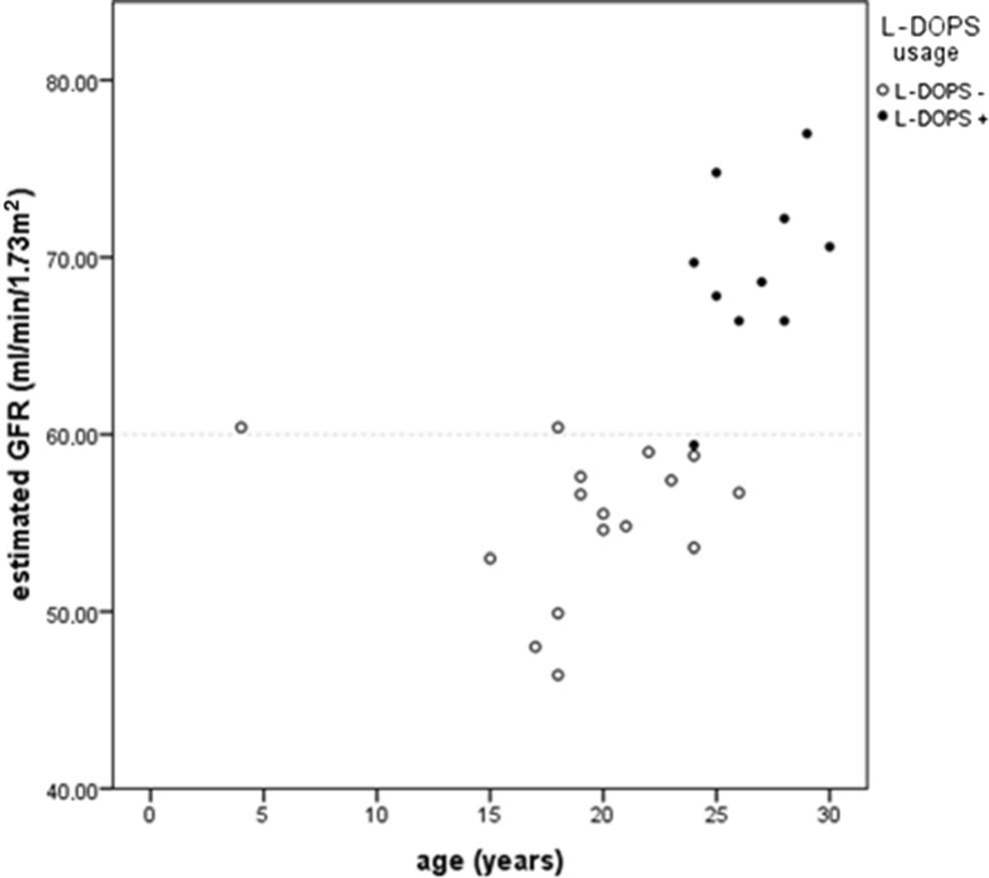
Repeated measurements of estimated glomerular filtration rate (eGFR; ml/min/1.73 m^2^) in our patient with DBH deficiency, showing decreased eGFR (<60 ml/min/1.73 m^2^. After she started on L-DOPS, her eGFR increased to between 68 and 78 ml/min/1.73 m^2^

Dopamine beta hydroxylase deficiency can be treated successfully by using L-threo-3,4-dihydroxyphenylserine (L-DOPS, droxidopa), which is converted directly to noradrenaline by aromatic L-amino acid decarboxylase (AADC), thereby bypassing the metabolic block (Fig. 1) [17, 18]. With L-DOPS treatment, patients no longer experience the symptoms associated with orthostatic hypotension [19], have an increased exercise tolerance, and markedly improved quality of life [20]. Our patient also achieved an impressive functional improvement after starting on L-DOPS. Furthermore, her eGFR showed a significant improvement, as is shown in Fig. 3. The negative effects of high dopamine levels on the kidney, therefore, whatever their exact mechanism, seemed to be reversible in this patient. The serum magnesium concentration before (0.64–0.77 mmol/L) and during (0.64–0.78 mmol/L) L-DOPS treatment remained in the low normal or subnormal range, with increased fractional urinary magnesium excretion (7.25–9.75 %, normally <4 %) during both states. The renal function study in this patient is a strong impetus for further detailed studies in more patients with DBH deficiency.
